# Supplementary material for: Development of a potent monoclonal antibody for treatment of human metapneumovirus infections
Source: Nat Commun. 2026 Feb 12;17:2714. doi: 10.1038/s41467-026-69328-w (PMC13013820; doi:10.1038/s41467-026-69328-w)

Supplemental Figure 1. Epitope binning of HMPV-neutralizing mAbs. Penta-HIS probes were coated with His-tagged GCN4-stabilized HMPV preF. The mAb listed first in the legend was loaded onto the coated probe followed by the mAb listed second in the legend. Cross-competition of 4F11 with site III mAb MxR (**a**) and with site Ø mAbs ADI-61026 and SAN32-2 (**b**). Binding measured by ELISA of 4F11 (**c**) or MxR (**d**) to wells coated with recombinant DS-CavEs2, v3B, or MPV-2c versions of HMPV preF. Data points are the average ± SD of three replicates for MPV-2c and two replicates for the remaining versions of HMPV preF. The dotted lines indicate data with recombinant versions of HMPV preF in which the amino acid at position 185 is the wild-type alanine.

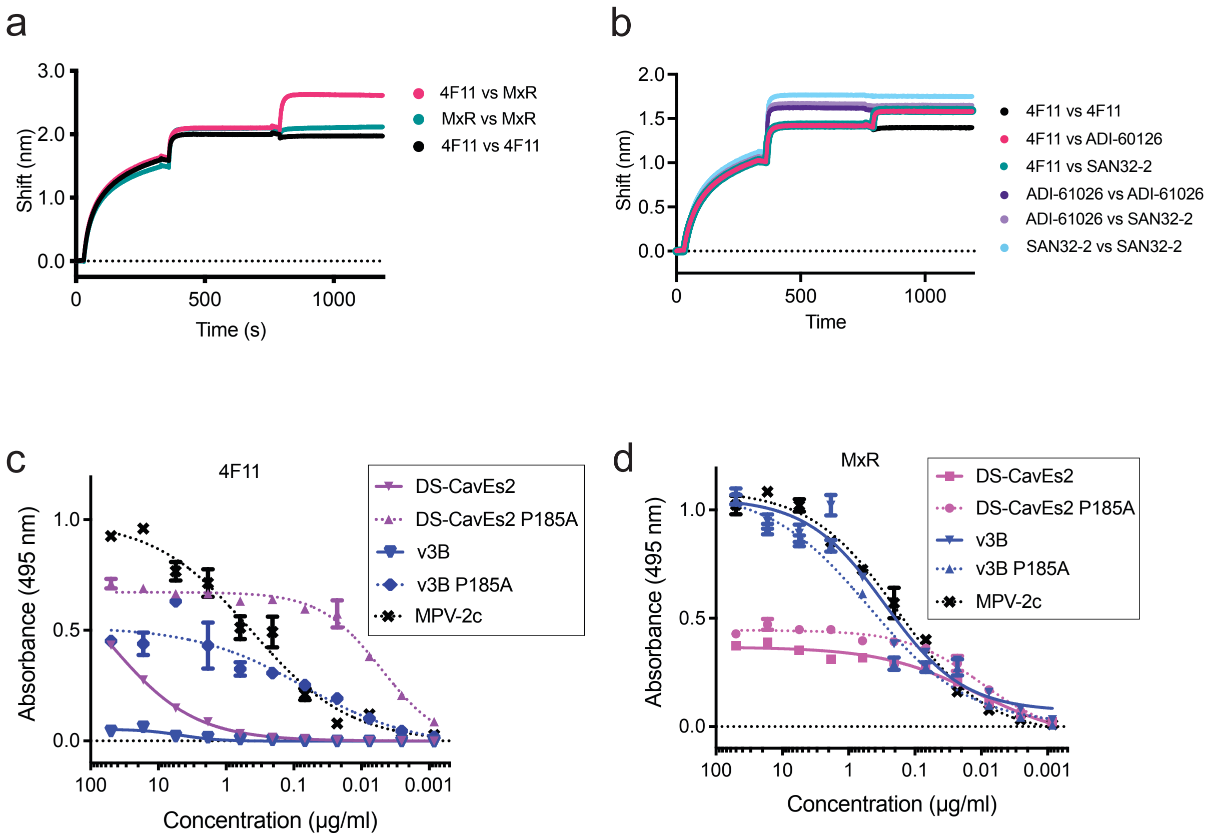

Supplemental Figure 2. Size exclusion chromatography (SEC) trace of DS-CavEs2 P185A trimer complexes. SEC trace of the complex consisting of a 3:1 ratio of MxR Fabs : trimer (blue) overlaid with the SEC trace of the complex consisting of a 1:1 ratio of 4F11 Fabs : trimer prebound to three MxR Fabs (orange).

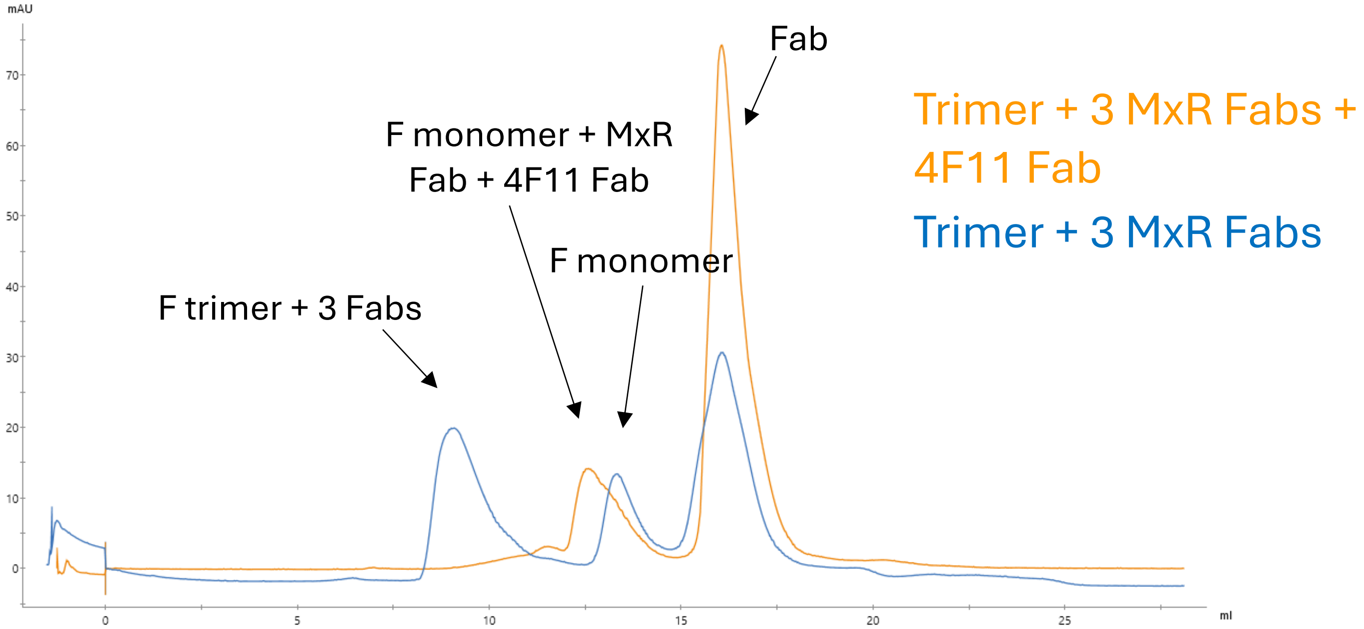

Supplemental Figure 3. CryoEM map processing and resolution. a) Processing pipeline for **Fig. 2b** map refinement of 4F11 and MxR Fabs complexed with DS-CavEs2 P185A monomer. **b)** Local resolution of the cryoEM map of 4F11 and MxR Fabs complexed with DS-CavEs2 P185A monomer. **c)** Particle orientation distribution of the cryoEM map of 4F11 and MxR Fabs complexed with DS-CavEs2 P185A monomer. **d)** Processing pipeline for **Fig. 2c** map refinement of 4F11 Fab bound to MPV-2c trimer. **e)** Local resolution of the cryoEM map of 4F11 Fab complexed with MPV-2c trimer. **f)** Particle orientation distribution of the cryoEM map of 4F11 Fab complexed with MPV-2c trimer.

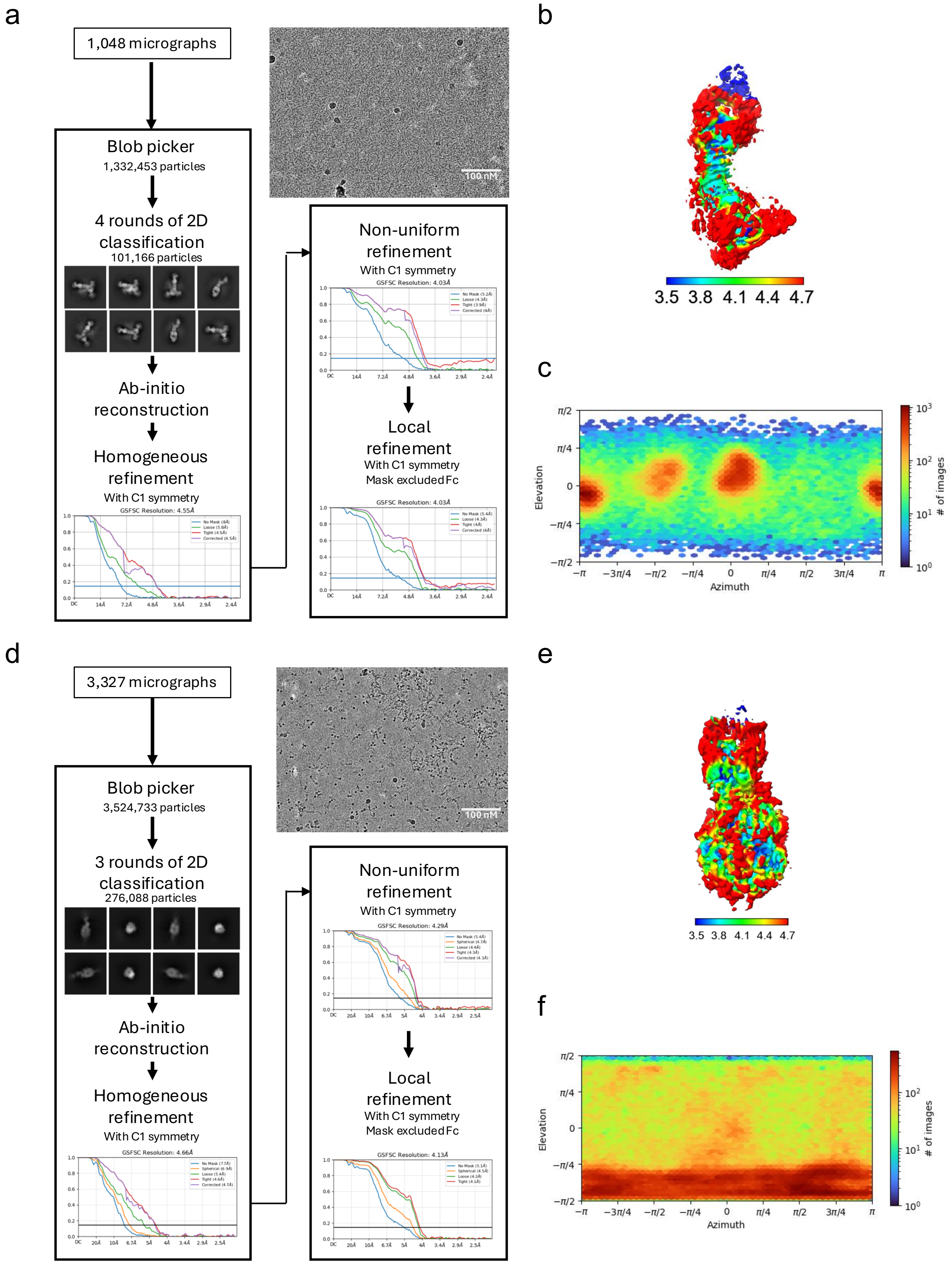
a
1,048 micrographs
100 nM
Blob picker
1,332,453 particles
4 rounds of 2D classification
101,166 particles
Ab-initio reconstruction
Homogeneous refinement
With C1 symmetry
GSFSC Resolution: 4.55Å
Non-uniform refinement
With C1 symmetry
GSFSC Resolution: 4.03Å
Local refinement
With C1 symmetry
Mask excluded Fc
GSFSC Resolution: 4.03Å
b
3.5 3.8 4.1 4.4 4.7
c
Elevation
Azimuth
# of images
d
3,327 micrographs
100 nM
Blob picker
3,524,733 particles
3 rounds of 2D classification
276,088 particles
Ab-initio reconstruction
Homogeneous refinement
With C1 symmetry
GSFSC Resolution: 4.66Å
Non-uniform refinement
With C1 symmetry
GSFSC Resolution: 4.29Å
Local refinement
With C1 symmetry
Mask excluded Fc
GSFSC Resolution: 4.13Å
e
3.5 3.8 4.1 4.4 4.7
f
Elevation
Azimuth
# of images

Supplemental Figure 4. Map around the interface of 4F11 and MPV-2c. a) Map (gray transparent surface) around site Ø (gray) from the side view (top panel) and top view (middle panel), and a zoom in of chain A of the F trimer (green) and chain B of the F trimer (yellow) that interact with 4F11 Fab (bottom panels). **b)** Map (gray transparent surface) around 4F11 Fab (purple) from the side view (top panel) and the paratope (middle panel). **c**) Map (gray transparent surface) around 4F11 Fab light chain (light purple) that contacts the N172 glycan (green) on the MPV-2c trimer (not shown) from three views.

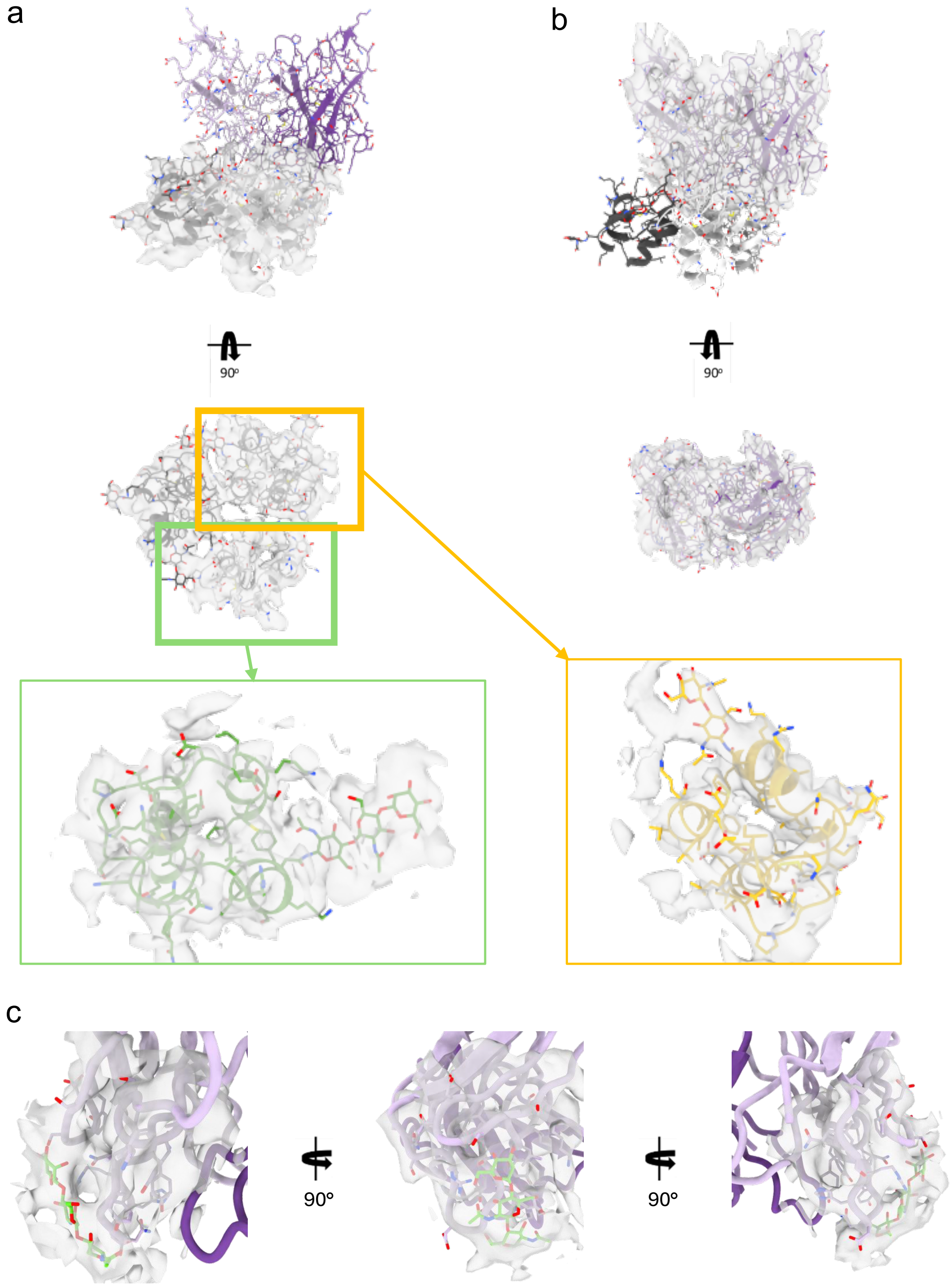

Supplemental Table 1. CryoEM structure statistics of 4F11 and MxR Fabs complexed with DS-CavEs2 P185A monomer and 4F11 Fab complexed with MPV-2c trimer.

	HMPV F monomer with 4F11 and MxR Fabs	MPV-2c with 4F11 Fab
**Data collection**		
Microscope	Glacios	Glacios
Voltage (kV)	200	200
Electron Dose (e-/$Å^2$)	60	60
Detector	K3	K3
Pixel Size (Å/px)	1.122	1.122
Defocus Range (µm)	-1.6 to -2.2	-1.6 to -2.2
Collection Tilt (°)	0	0 and 27
Magnification	92,000x	92,000x
**Reconstruction**		
Software	CryoSPARC v4.4	CryoSPARC v4.4
Selected Micrographs	1,048	3,432
Selected Particles	101,166	276,088
Symmetry	C1	C1
Box Size (px)	256	256
Resolution (Å) ($FSC_{0.143}$)	4.03	4.13
**Refinement**		
Map B factor ($Å^2$)	244.7	194.8
No. atoms	n.d.	11934
Protein	n.d.	1547
Water	n.d.	0
Ligand	n.d.	8
Mean B-factor (Å)		
Protein	n.d.	90.87
Water	n.d.	0.00
Ligand	n.d.	65.90
RMS bond length (Å)	n.d.	0.012
RMS bond angle (°)	n.d.	2.307
**Validation**		
MolProbity	n.d.	1.48
Clashscore	n.d.	3.65
CaBLAM outliers (%)	n.d.	3.04
EMRinger	n.d.	1.56
Rotamer Outliers (%)	n.d.	0.46
Ramachandran		
Favored (%)	n.d.	95.36
Disallowed (%)	n.d.	0.33
**PDB ID**	n.d.	pdb_00009ORE
**EMDB ID**	EMD-70774	EMD-70773

*n.d.: not deposited

Supplemental Figure 5. Models of two and three 4F11 Fabs bound to the MPV-2c trimer. a) Model of two 4F11 Fabs bound to the MPV-2c trimer from a side view (left), top view (middle), and top view with the F trimer hidden (right). **b)** Model of three 4F11 Fabs bound to the MPV-2c trimer from a side view (left), top view (middle), and top view with the F trimer hidden (right). The purple Fab is in the cryoEM structure and the blue and green Fabs are the modelled second and third Fabs, respectively. Clashes of the Fabs are shown in red.

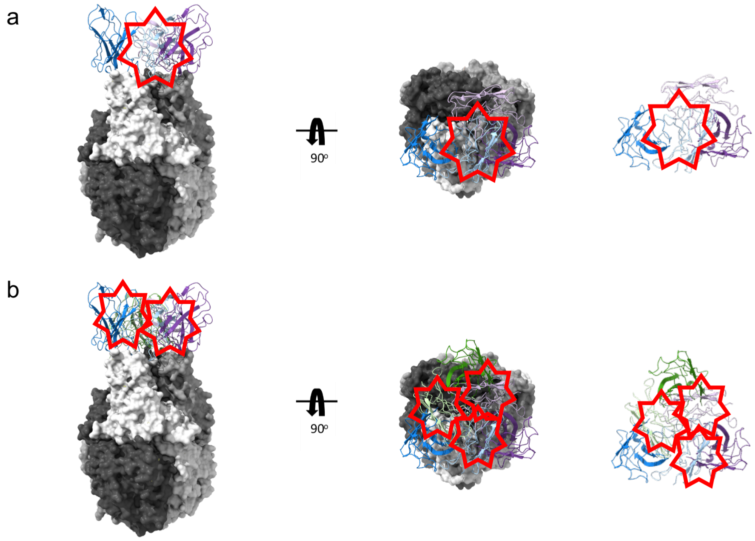

Supplemental Figure 6. Comparison of binding to the MPV-2c trimer (gray) by 4F11 Fab (purple) and M8C10 Fab (pink).

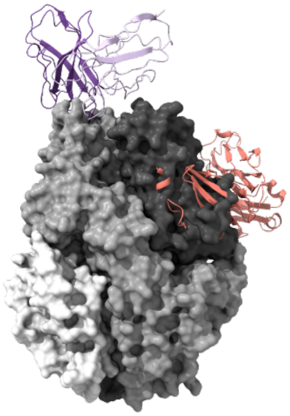

Supplemental Figure 7. Buried surface area of MPV-2c and 4F11. a) BSA plot of protomers A and B of MPV-2c when interacting with 4F11 VH (dark purple for interactions with protomer A) and VL (light purple for interactions with Protomer A and green for interactions with protomer B). Sequences of MPV-2c F and four HMPV subtypes (A1 accession ID KC562236, A2 accession ID PP315925, B1 accession ID OP904070, and B2 accession ID PV052229) and RSV F are shown and aligned underneath. **b)** BSA plot of 4F11 VH (top) when interacting with MPV-2c protomer A (dark purple). The 4F11 VH and germline gene VH5-51*01 sequences are aligned on the x-axis. BSA plot of 4F11 VL (bottom) when interacting with MPV-2c protomer A (light purple), protomer B (green), and glycan of protomer A (orange). The 4F11 VL and germline gene VK2-28*01 sequences are aligned on the x-axis. The CDRs of 4F11 VH and VL are highlighted in red while the framework regions are in black.

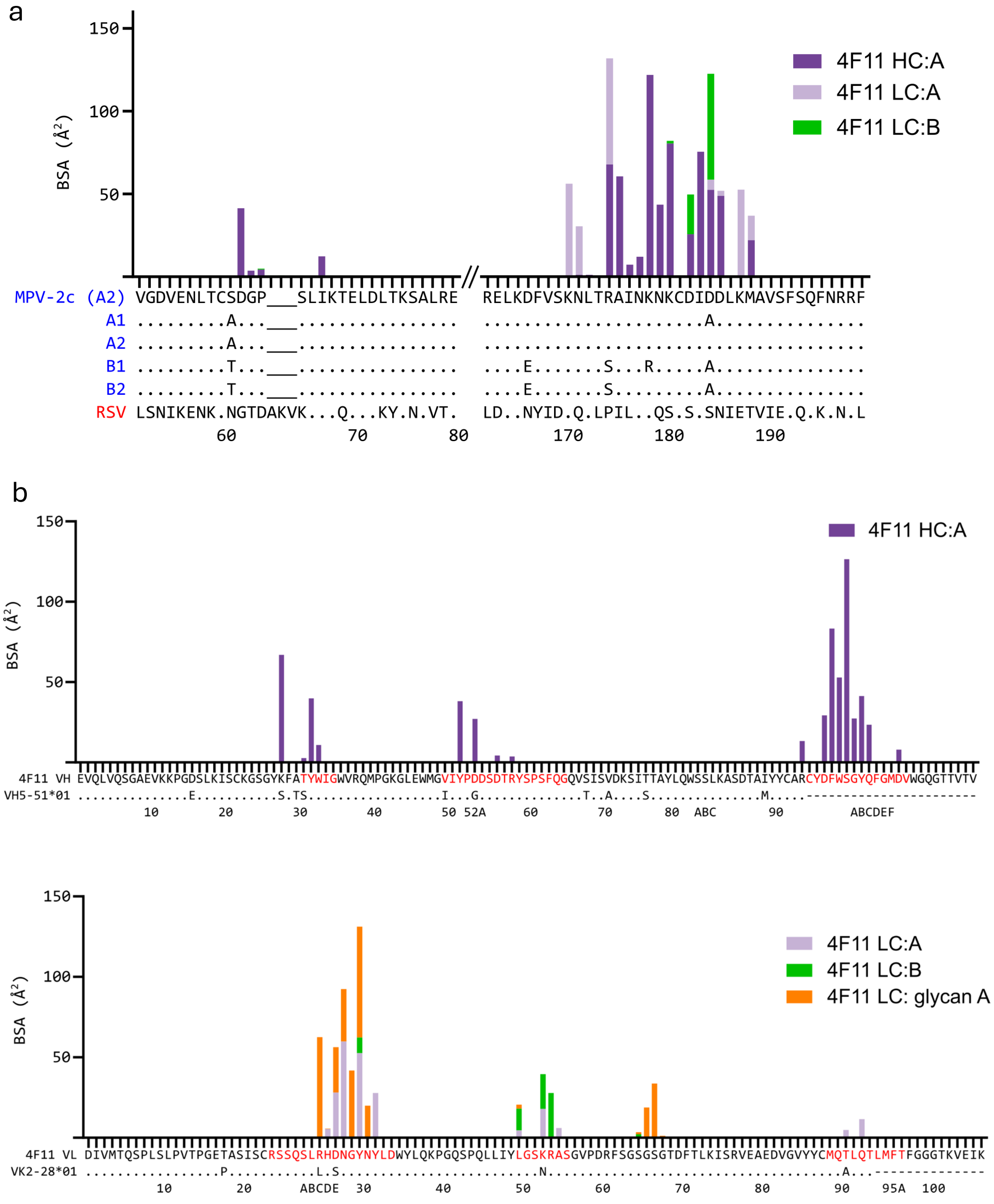

Supplemental Figure 8. Binding kinetics and affinity of 4F11 as measured by biolayer interferometry. 4F11 Fab was loaded onto FAB2G probes, and binding was measured with serial dilutions of HMPV F monomer (left) or the MPV-2c F trimer (right) to determine binding kinetics (bottom). Dotted black lines represent fitted curves using a 1:1 model. Values reported below include the average K_D, average k_{dis}, and average k_{on} for each binding curve.

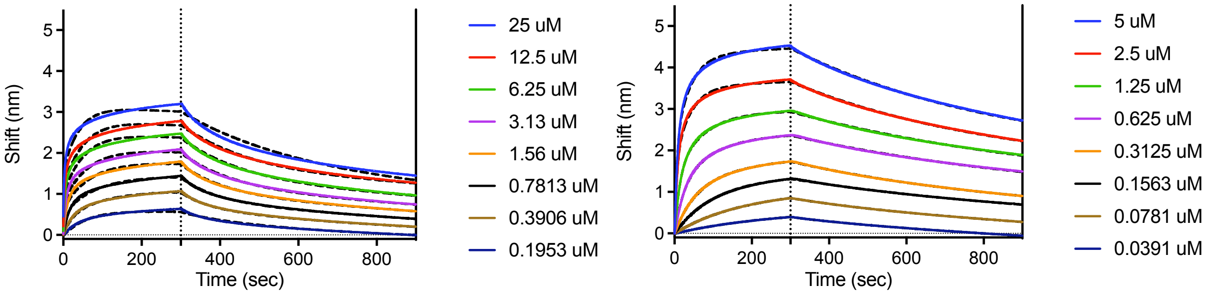

	K_D (M)	K_D error	k_{on}(1/Ms)	k_{on} error	k_{dis}(1/s)	k_{dis} error	Full R^2
Monomer	1.01E-07	2.37E-09	2.71E+04	4.15E+02	2.04E-03	1.89E-05	0.97
Trimer	2.45E-08	4.37E-10	4.06E+04	4.87E+02	9.95E-04	4.90E-06	0.99

Supplemental Figure 9. Binding of 4F11 (purple) to the prefusion MPV-2c F trimer (gray) compared to the postfusion F trimer (green, PDB ID 5L1X). Clashes between 4F11 Fab and postfusion F are circled in red.

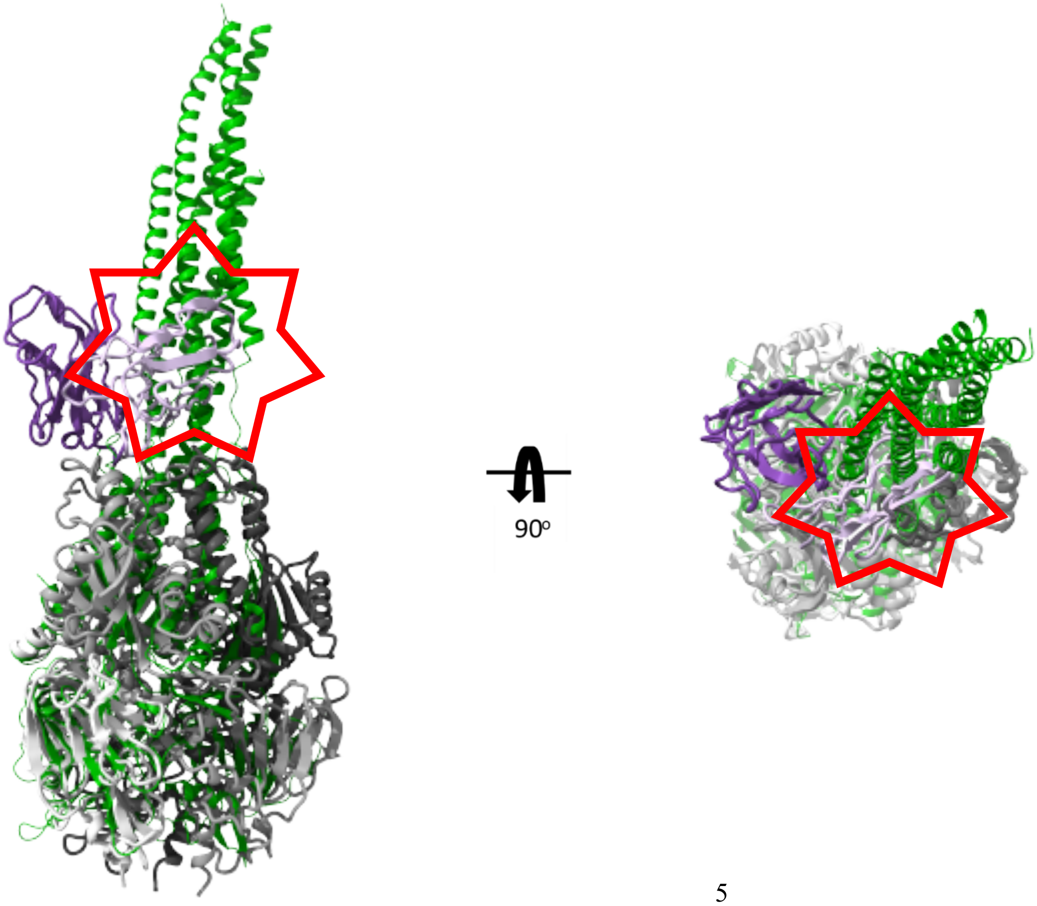

Supplemental Figure 10. Binding of 4F11 to HMPV F with enzymatic removal of glycans and to RSV F. a) HMPV F monomer was produced in GnTI$^-$ cells and treated overnight with EndoH to remove glycans. Undigested or EndoH-treated HMPV F monomer was loaded onto penta-HIS probes and biolayer interferometry was performed to measure association with 4F11 Fab. **b)** 4F11 or MxR was loaded onto anti-human capture probes and biolayer interferometry was performed to measure binding to RSV preF.

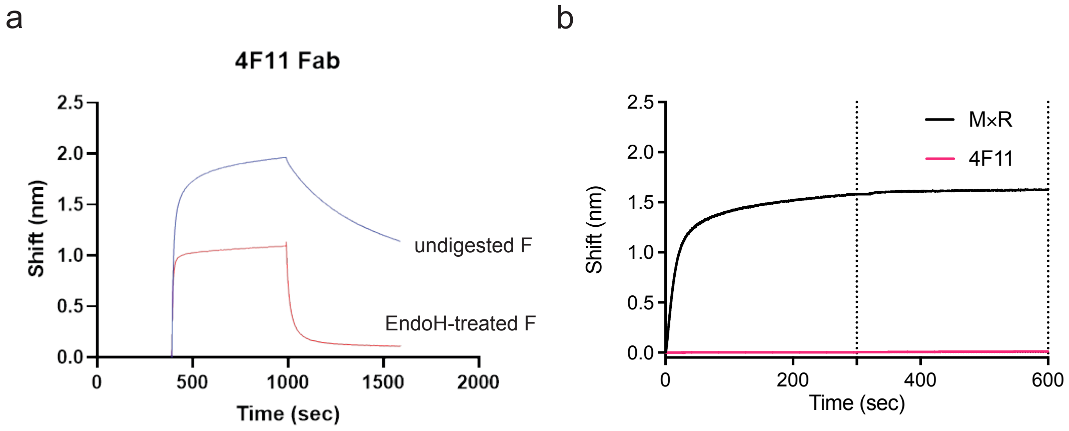

Supplemental Figure 11. Comparison of the 4F11 epitope with other site Ø mAbs. Comparison of 4F11 Fab (heavy chain: purple; light chain: light purple) with SAN32-2 Fab (heavy chain, orange; light chain, light orange) (left) from PDB 7TL0 and ADI-61026 Fab (heavy chain, red; light chain, light red) (right) from PDB 8CW9 binding to the MPV-2c trimer (shades of gray).

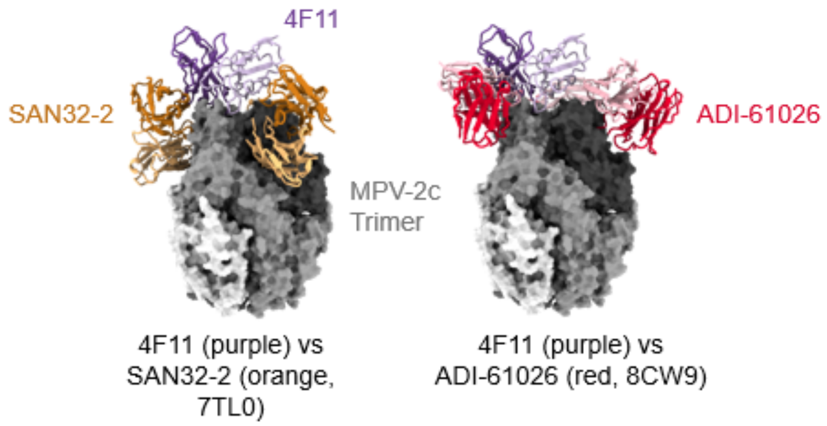

Supplemental Figure 12. Phylogenetic analysis and geographic distribution of HMPV F sequences based on amino acid sequence at position 179. a) Phylogenetic tree of HMPV sequences visualized using Nextstrain. Colors indicate genotypic variations at codon 179 of the F protein, and branch lengths represent time. **b**) Geographic distribution of HMPV sequences visualized using Nextstrain. Colors in each circle represent genotype variations at amino acid 179 of the F protein, with placement based on sequence metadata. Data sourced from publicly available sequences analyzed by Nextstrain (https://nextstrain.org/hmpv). Accessed February 12, 2025, with attribution to nextstrain.org, and without any changes made. Open-source license AGPL-3.0 [https://opensource.org/license/agpl-v3] and MIT [https://opensource.org/license/MIT].

a

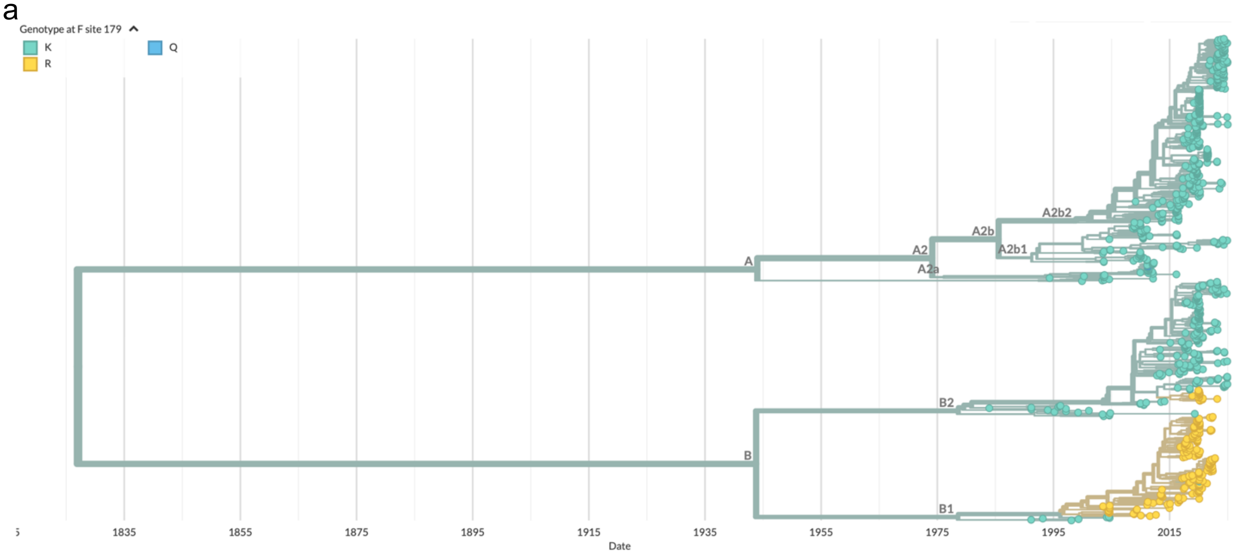

b

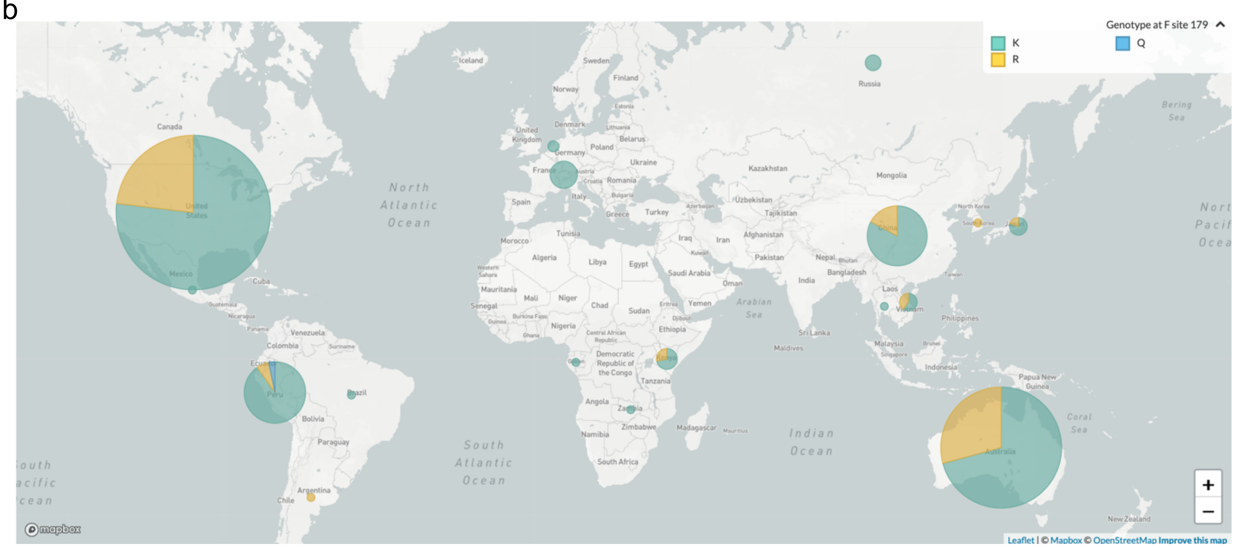

Supplemental Figure 13. **Phylogenetic analysis and geographic distribution of HMPV F sequences based on amino acid sequence at position 172**. **a**) Phylogenetic tree of HMPV sequences visualized using Nextstrain. Colors indicate genotypic variations at codon 172 of the F protein, and branch lengths represent time. **b**) Geographic distribution of HMPV sequences visualized using Nextstrain. Colors in each circle represent genotypic variations at amino acid 172 of the F protein, with placement based on sequence metadata. Data sourced from publicly available sequences analyzed by Nextstrain (https://nextstrain.org/hmpv). Accessed February 12, 2025, with attribution to nextstrain.org, and without any changes made. Open-source license AGPL-3.0 [https://opensource.org/license/agpl-v3] and MIT [https://opensource.org/license/MIT].

a

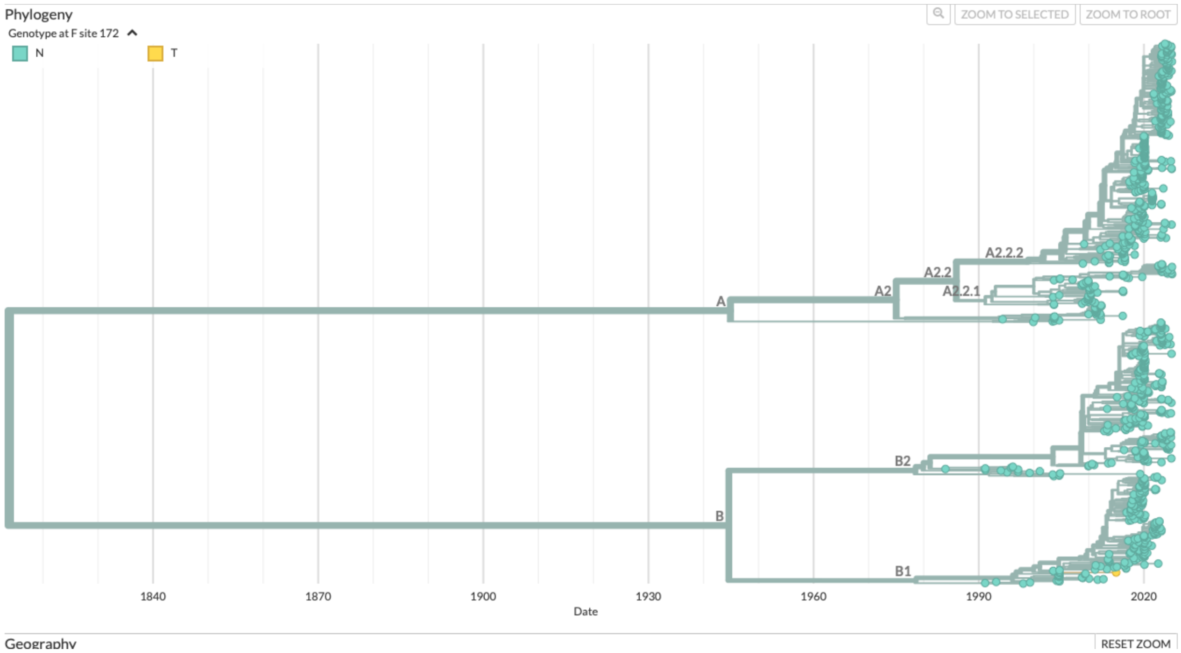

b

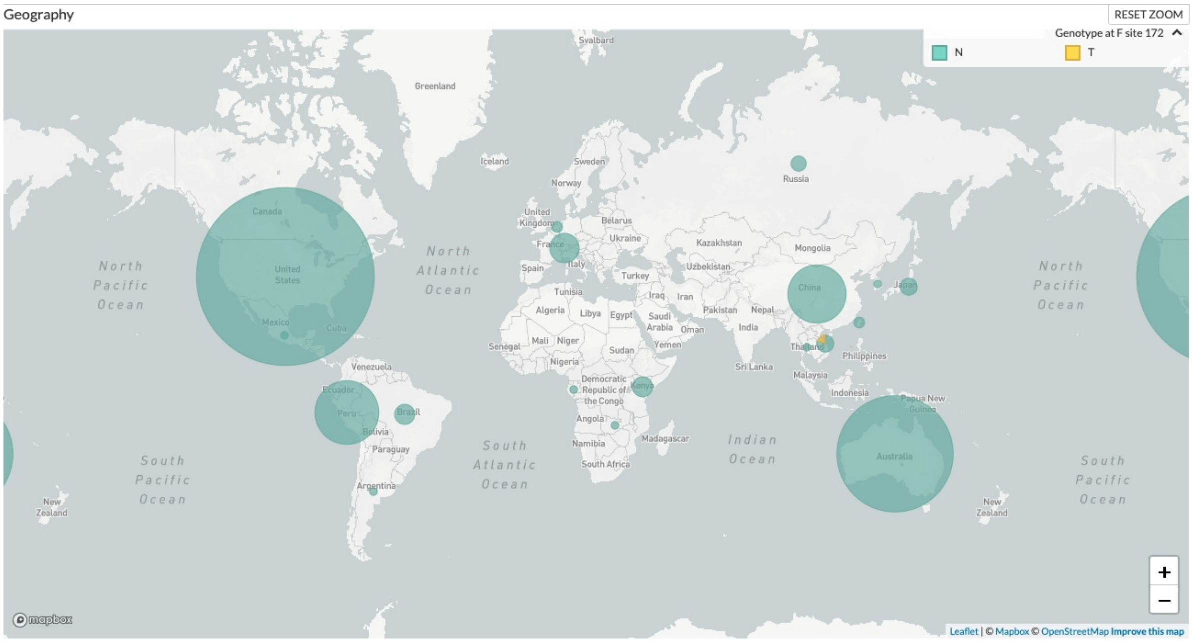

Supplement: Supplementary file 1 — Supplementary Information [file 41467_2026_69328_MOESM1_ESM.pdf]
