# Supplementary material for: Development of a potent monoclonal antibody for treatment of human metapneumovirus infections
Source: Nat Commun. 2026 Feb 12;17:2714. doi: 10.1038/s41467-026-69328-w (PMC13013820; doi:10.1038/s41467-026-69328-w)
Supplement: Supplementary file 2 — Reporting Summary [file 41467_2026_69328_MOESM2_ESM.pdf]

Corresponding author(s): Jim Boonyaratanakornkit
Last updated by author(s): Nov 15, 2025

Reporting Summary

Nature Portfolio wishes to improve the reproducibility of the work that we publish. This form provides structure for consistency and transparency in reporting. For further information on Nature Portfolio policies, see our Editorial Policies and the Editorial Policy Checklist.

Statistics

For all statistical analyses, confirm that the following items are present in the figure legend, table legend, main text, or Methods section.

n/a	Confirmed	
☐	☒	The exact sample size (*n*) for each experimental group/condition, given as a discrete number and unit of measurement
☐	☒	A statement on whether measurements were taken from distinct samples or whether the same sample was measured repeatedly
☐	☒	The statistical test(s) used AND whether they are one- or two-sided<br>*Only common tests should be described solely by name; describe more complex techniques in the Methods section.*
☐	☒	A description of all covariates tested
☒	☐	A description of any assumptions or corrections, such as tests of normality and adjustment for multiple comparisons
☐	☒	A full description of the statistical parameters including central tendency (e.g. means) or other basic estimates (e.g. regression coefficient) AND variation (e.g. standard deviation) or associated estimates of uncertainty (e.g. confidence intervals)
☐	☒	For null hypothesis testing, the test statistic (e.g. *F*, *t*, *r*) with confidence intervals, effect sizes, degrees of freedom and *P* value noted<br>*Give P values as exact values whenever suitable.*
☒	☐	For Bayesian analysis, information on the choice of priors and Markov chain Monte Carlo settings
☐	☒	For hierarchical and complex designs, identification of the appropriate level for tests and full reporting of outcomes
☒	☐	Estimates of effect sizes (e.g. Cohen's *d*, Pearson's *r*), indicating how they were calculated

Our web collection on statistics for biologists contains articles on many of the points above.

Software and code

Policy information about availability of computer code

Data collection	Diva 9.0; Octet Data Analysis Software Release 9.0; Serial EM 4.2
Data analysis	Flowjo 11; GraphPad Prism 10.6.1; ImageJ 1.54r; cryoSPARC v4.3.1; Phenix software suite, Coot; ChimeraX 1.10.1; fastp v0.23; Snakemake (v8.14) pipeline (available at https://github.com/jbloomlab/Virus-Antibody-Passaging/tree/main); Nextstrain

For manuscripts utilizing custom algorithms or software that are central to the research but not yet described in published literature, software must be made available to editors and reviewers. We strongly encourage code deposition in a community repository (e.g. GitHub). See the Nature Portfolio guidelines for submitting code & software for further information.

Data

Policy information about availability of data

All manuscripts must include a data availability statement. This statement should provide the following information, where applicable:

- Accession codes, unique identifiers, or web links for publicly available datasets
- A description of any restrictions on data availability
- For clinical datasets or third party data, please ensure that the statement adheres to our policy

Sequencing and structural data that support the findings of this study have been deposited in the Protein Data Bank (PDB) and Electron Microscopy Data Bank (EMDB) and are accessible through accession numbers 9ORE, EMD-70773, and EMD-70774. Source data are provided with this paper.

Research involving human participants, their data, or biological material

Policy information about studies with human participants or human data. See also policy information about sex, gender (identity/presentation), and sexual orientation and race, ethnicity and racism.

Reporting on sex and gender	Data on sex and gender were not available and are not reported.
Reporting on race, ethnicity, or other socially relevant groupings	Data on race, ethnicity, or social groupings were not available and are not reported.
Population characteristics	Coded human cells were derived from peripheral blood and spleen. Blood samples were obtained from the Seattle Area Control Study. This is an observational cohort of healthy adults living in the Seattle area (Fred Hutch IRB #5567). The blood samples were de-identified. Spleen samples were deemed non-human subjects research by the Fred Hutch IRB (Fred Hutch #0020731) and as defined by the Common Rule from the Office for Human Research Protections. Tissue was de-identified and originated from deceased donors in which the spleen would have otherwise been discarded during procurement of other organs (i.e., liver) for donation. There was no engagement with the individuals from which samples originated. Therefore, the research met the criteria for non-human subjects research.
Recruitment	Participants in the Seattle Area Control observational cohort were recruited through local advertisements to collect blood from healthy HIV-seronegative individuals as part of the HIV Vaccine Trials Network. Informed consent was obtained before enrollment. Potential self-selection bias may be present towards individuals who are more likely to participate in research trials and donate blood. This bias is expected to have little to no impact on the study results.
Ethics oversight	Fred Hutch IRB

Note that full information on the approval of the study protocol must also be provided in the manuscript.

Field-specific reporting

Please select the one below that is the best fit for your research. If you are not sure, read the appropriate sections before making your selection.

[x] Life sciences   [ ] Behavioural & social sciences   [ ] Ecological, evolutionary & environmental sciences

For a reference copy of the document with all sections, see nature.com/documents/nr-reporting-summary-flat.pdf

Life sciences study design

All studies must disclose on these points even when the disclosure is negative.

Sample size	Sample sizes for neutralization assays and animal challenge experiments are consistent with or exceed the sample sizes generally accepted by the field based on published work (PMID 29768937; PMID 26367224; PMID 7049952; PMID 17362988).
Data exclusions	No data was excluded from the analysis.
Replication	Neutralization and animal challenge experiments were repeated independently at least twice to ensure reproducibility. Each animal challenge experiment consisted of 5-9 animals per group. All attempts at replication were successful.
Randomization	Hamsters were obtained from Charles River Laboratory and were matched for age and sex. After receipt, hamsters were randomly assigned to either experimental or control groups.
Blinding	Experimental techniques were not blinded, since the hamster challenge experiments were not designed as a randomized controlled trial and dosing of animals needed to be verified. The harvesting and processing of respiratory tissue also needed to be performed unblinded in order to avoid cross-contamination between virus-infected tissue and control tissue.

Reporting for specific materials, systems and methods

We require information from authors about some types of materials, experimental systems and methods used in many studies. Here, indicate whether each material, system or method listed is relevant to your study. If you are not sure if a list item applies to your research, read the appropriate section before selecting a response.

Materials & experimental systems

n/a	Involved in the study	
☐	☒	Antibodies
☐	☒	Eukaryotic cell lines
☒	☐	Palaeontology and archaeology
☐	☒	Animals and other organisms
☒	☐	Clinical data
☒	☐	Dual use research of concern
☒	☐	Plants

Methods

n/a	Involved in the study	
☒	☐	ChIP-seq
☐	☒	Flow cytometry
☒	☐	MRI-based neuroimaging

Antibodies

Antibodies used

4F11, 4E11, and 3B5 are the monoclonal antibodies described in this paper. Antibodies for flow cytometry include: anti-CD19 BUV39S (SJ2SC1, BD, cat#S635S1, 1:20 dilution), anti-CD3 BV711 (UCHTl, BD, cat#S6372S, 1:50 dilution), anti-CD14 BV711 (M0P-9, BD, cat#S63372, 1:50 dilution), anti-CD16 BV711 (3G8, BD, cat#S63127, 1:50 dilution), and anti-lgD BV60S (IA6-2, BD, cat#S63313, 1:50 dilution). MxR (Caban et. al., Nat Commun, 2023), ADI-61026 (Rappazzo et. al., Immunity, 2022), and SAN32-2 (Rush et. al., Cell Rep, 2022) were produced based on previously published data.

Validation

Flow cytometry antibodies were obtained commercially and have been validated by their manufacturers with specificity testing of 1-3 target cell types in single or multi-color analysis (including positive and negative cell types). The antibodies were also serially diluted to determine the optimal concentration for flow cytometry. Other antibodies were validated by verifying binding to cognate antigens by biolayer interferometry and neutralization of target viruses by plaque reduction neutralization assays.

Eukaryotic cell lines

Policy information about cell lines and Sex and Gender in Research

Cell line source(s)

293F cells (Thermo Fisher, cat#R79007); Vero cells (ATCC CCL-81); LLC-MK2 cells (ATCC CCL-7.1); 3T3 CD40L/IL2/IL21 feeder cells were obtained from Dr. Andrew McGuire at the Fred Hutchinson Cancer Center and are derived from the Division of AIDS, NIAID, NIH (Cat#12S3S).

Authentication

Cell lines from Thermo Fisher and ATCC have been authenticated by the manufacturer. Cell lines were also authenticated by microscopic morphology examination upon thawing and at each passage. 3T3 CD40L/IL2/IL21 feeder cells have been screened for the continued expression of CD40L by flow cytometry and the secretion of cytokines by ELISA.

Mycoplasma contamination

Cell lines were tested regularly for mycoplasma contamination using the MycoProbe kit. All testing was negative for this study.

Commonly misidentified lines
(See ICLAC register)

n/a

Animals and other research organisms

Policy information about studies involving animals; ARRIVE guidelines recommended for reporting animal research, and Sex and Gender in Research

Laboratory animals

Golden Syrian hamsters, 4-8 weeks of age

Wild animals

This study did not involve wild animals.

Reporting on sex

Animal challenge experiments were conducted with male hamsters. An association between sex and clinical outcomes has not been observed in human adults with HMPV. Therefore, we did not anticipate an effect of sex on the endpoints in this study. Female hamsters also are extremely territorial and cannot not be co-housed due to fighting, which can lead to serious injury and death.

Field-collected samples

This study did not involve field-collected samples.

Ethics oversight

All work with animals was reviewed and approved by the Fred Hutch Institutional Animal Care and Use Committee.

Note that full information on the approval of the study protocol must also be provided in the manuscript.

Plants

Seed stocks

Report on the source of all seed stocks or other plant material used. If applicable, state the seed stock centre and catalogue number. If plant specimens were collected from the field, describe the collection location, date and sampling procedures.

Novel plant genotypes

Describe the methods by which all novel plant genotypes were produced. This includes those generated by transgenic approaches, gene editing, chemical/radiation-based mutagenesis and hybridization. For transgenic lines, describe the transformation method, the number of independent lines analyzed and the generation upon which experiments were performed. For gene-edited lines, describe the editor used, the endogenous sequence targeted for editing, the targeting guide RNA sequence (if applicable) and how the editor was applied.

Authentication

Describe any authentication procedures for each seed stock used or novel genotype generated. Describe any experiments used to assess the effect of a mutation and, where applicable, how potential secondary effects (e.g. second site T-DNA insertions, mosiacism, off-target gene editing) were examined.

Flow Cytometry

Plots

Confirm that:

- [x] The axis labels state the marker and fluorochrome used (e.g. CD4-FITC).
- [x] The axis scales are clearly visible. Include numbers along axes only for bottom left plot of group (a 'group' is an analysis of identical markers).
- [x] All plots are contour plots with outliers or pseudocolor plots.
- [x] A numerical value for number of cells or percentage (with statistics) is provided.

Methodology

Sample preparation

Single cells suspensions of human peripheral blood mononuclear cells or spleen were thawed into DMEM with 10% fetal calf serum and 100 U/ml penicillin plus 100 µg/ml streptomycin. Cells were centrifuged and resuspended in 50 µL of ice-cold FACS buffer composed of PBS and 1% newborn calf serum (Thermo Fisher). His tag APC/Dylight755- and His tag PE/Dylight650-conjugated tetramers were added at a final concentration of 25 nM in the presence of 2% rat and mouse serum (Thermo Fisher) and incubated at room temperature for 10 min. PreF APC or PE tetramers were then added at a final concentration of 5 nM and incubated on ice for 25 min, followed by a 10 mL wash with ice-cold FACS buffer. Each sample was incubated with 50 µL of anti-APC- or anti-PE-conjugated microbeads (Miltenyi Biotec) on ice for 25 min. 5 mL of FACS buffer was added, and this mixture was passed over a magnetized LS column (Miltenyi Biotec). The column was washed once with 5 mL ice-cold FACS buffer and then removed from the magnetic field and 5 mL ice-cold FACS buffer was pushed through the unmagnetized column twice using a plunger to elute the bound cell fraction. The bound and flowthrough fractions were incubated in 50 µL of FACS buffer containing a cocktail of antibodies for 25 minutes on ice prior to washing and analysis on a FACS Aria (BD).

Instrument

FACS Aria II (BD Bioscience)

Software

Diva 9; Flowjo 11

Cell population abundance

Post-sort populations of preF-binding cells were > 50% positive for IgG production. Three monoclonal antibodies cloned from these B cells were tested for neutralization of HMPV by a plaque reduction neutralization assay.

Gating strategy

For all flow cytometry experiments, cells in the lymphocyte gate of FSC-A/SSC-A were used for analysis. Single cells were identified via SSC-A vs SSC-W. Live B cells were identified by gating FVD-/CD3-/CD14-/CD16-/CD19+/IgD- cells. Specific figures were subsequently gated as follows:
Figure lb: His tag- and HMPV preF+/RSV preF-.

- [x] Tick this box to confirm that a figure exemplifying the gating strategy is provided in the Supplementary Information.
